# Proper Interpretation of Dissolved Nitrous Oxide Isotopes, Production Pathways, and Emissions Requires a Modelling Approach

**DOI:** 10.1371/journal.pone.0090641

**Published:** 2014-03-07

**Authors:** Simon J. Thuss, Jason J. Venkiteswaran, Sherry L. Schiff

**Affiliations:** Department of Earth and Environmental Sciences, University of Waterloo, Waterloo, Ontario, Canada; Scottish Association for Marine Science, United Kingdom

## Abstract

Stable isotopes (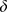 ^15^N and 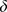 ^18^O) of the greenhouse gas N_2_O provide information about the sources and processes leading to N_2_O production and emission from aquatic ecosystems to the atmosphere. In turn, this describes the fate of nitrogen in the aquatic environment since N_2_O is an obligate intermediate of denitrification and can be a by-product of nitrification. However, due to exchange with the atmosphere, the 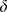 values at typical concentrations in aquatic ecosystems differ significantly from both the source of N_2_O and the N_2_O emitted to the atmosphere. A dynamic model, SIDNO, was developed to explore the relationship between the isotopic ratios of N_2_O, N_2_O source, and the emitted N_2_O. If the N_2_O production rate or isotopic ratios vary, then the N_2_O concentration and isotopic ratios may vary or be constant, not necessarily concomitantly, depending on the synchronicity of production rate and source isotopic ratios. Thus *prima facie* interpretation of patterns in dissolved N_2_O concentrations and isotopic ratios is difficult. The dynamic model may be used to correctly interpret diel field data and allows for the estimation of the gas exchange coefficient, N_2_O production rate, and the production-weighted 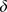 values of the N_2_O source in aquatic ecosystems. Combining field data with these modelling efforts allows this critical piece of nitrogen cycling and N_2_O flux to the atmosphere to be assessed.

## Introduction

Nitrous oxide (N_2_O) is a powerful greenhouse gas, 298 times more potent than CO_2_ over a 100-year time line [1]. Atmospheric N_2_O concentrations have been increasing at a rate of 0.25%/year over the last 150 years [2]. Consequently, the global N_2_O budget has been the subject of intensive research efforts over the past few decades. N_2_O is produced through multiple microbial pathways: hydroxylamine oxidation during nitrification and as an obligate intermediate during denitrification and nitrifier–denitrification. Because these pathways of N_2_O production have different stable isotopic enrichment factors, isotopic analysis of N_2_O can potentially distinguish N_2_O produced through different pathways or from different sources [3]. Identifying N_2_O sources will provide insights on the fate of N at the ecosystem-scale (e.g., [4]–[6]). The isotopic ratios of N_2_O produced in soil environments (e.g., [7]–[11]), and in aquatic environments (e.g., [12]–[18]) have been measured to some extent. Although N_2_O production in rivers and estuaries is a significant portion of the global N_2_O budget (approximately 1.5 TgN/year, [19]), few studies report isotopic data for rivers [5], [20], [21].

In ice-free aquatic ecosystems, the 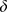 ^15^N and 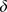 ^18^O of dissolved N_2_O is affected by gas exchange with the atmosphere. As a result, the isotopic ratios of dissolved N_2_O are not equal to those of the N_2_O produced within the aquatic ecosystem and continue to change as atmospheric exchange (both ingassing and outgassing) occurs. In addition, isotopic fractionation during influx and efflux causes the isotopic ratios of N_2_O flux emitted to the atmosphere to be different than that of the dissolved N_2_O [22]. Thus, the simple method of calculating the instantaneous isotopic ratios of the N_2_O flux by taking measured dissolved isotopic ratios, adding an equilibrium isotope fractionation, and applying them to measured flux rates is inappropriate. Adjustments of measured isotopic ratios are necessary to understand the isotopic ratios of both produced and emitted N_2_O.

In this paper, we present a dynamic model of the stable isotopic composition of both the dissolved and emitted N_2_O in aqueous systems. We apply this model to two different measured diel patterns of the isotopic ratios of N_2_O in an aquatic ecosystem. We use the model to elucidate the relationship between the isotopic ratios of source, dissolved, and emitted N_2_O, to allow for improved interpretation of dissolved N_2_O isotope data. Ultimately, a process-based understanding on N cycling with aquatic ecosystems may be developed based on interpretation of N cycling processes.

## Materials and Methods

### Stable Isotopes of N_2_O

N_2_O is an asymmetric molecule: the most abundant isotopologues of N_2_O are 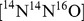, 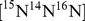, 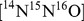 and 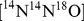. The isotopic ratios, ^15^N: ^14^N and ^18^O: ^16^O, are:
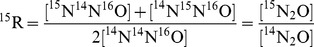
(1)




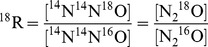
(2)where 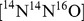, 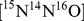, 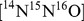 and 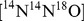 represent the concentrations of the various N_2_O isotopologues. Note that 15R is the bulk ^15^N: ^14^N ratio and represents an average ratio of the two ^15^N isotopomers and isotopic ratios are reported as 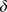 ^15^N relative to air and 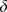 ^18^O relative to VSMOW. Although the isotopic ratio of the ^15^N isotopomers can be measured (e.g., [23]–[25]), the gas exchange fractionation factors are not affected by the intramolecular distribution of ^15^N [22]. Many laboratories cannot measure the intramolecular distribution of ^15^N and analysis of the bulk ^15^N: ^14^N ratio of N_2_O is more common [26]. Here, we confine our analysis to bulk ^15^N: ^14^N ratios and use ^15^N_2_O to represent the average abundance of the two ^15^N isotopomers. The same approach could easily be extended to consider each isotopologue separately.

### Dynamic Isotope Model for Dissolved N_2_O

A simple three box model (SIDNO, Stable Isotopes of Dissolved Nitrous Oxide) was created using Stella modelling software (version 9.1.4, http://www.iseesystems.com) in order to study the relationships between the isotopic ratios of source, dissolved and emitted N_2_O (model file is available at https://github.com/jjvenky/SIDNO and by contacting the corresponding author). This model is an adaptation of the isotopic gas exchange portion of the PoRGy model [27], which successfully modelled diel isotopic ratios of O_2_ resulting from photosynthesis, respiration, and gas exchange in aquatic ecosystems. One key difference is photosynthetically produced O_2_ in PoRGy has a 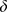 ^18^O value fixed by the H_2_O molecules, whereas SIDNO has N_2_O production 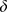 ^15^N and 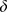 ^18^O values that can vary independently of each other and of N_2_O production rate in order to simulate variability in nitrification and denitrification.

One box in SIDNO is used for the total mass of dissolved N_2_O and two additional boxes for the dissolved masses of the two heavy isotopologues (^15^N_2_O and N_2_^18^O). The boxes are open to the atmosphere for gas exchange, are depth agnostic, and each box can gain N_2_O via a production term; there is no N_2_O consumption term since the 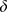 values of N_2_O are largely controlled by the production pathways [28], [29] though certain waters can exhibit significant N_2_O reduction to N_2_ [30], [31]. The masses and magnitude of the flows of ^15^N_2_O and N_2_^18^O relative to bulk N_2_O are used to calculate the isotopic composition of source, dissolved, and emitted N_2_O. Although isotopic ratios are used in the model, we discuss 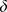 values that are common for reporting isotopic ratios. N_2_O production rate and its 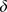 values are user-defined and can be adjusted for diel patterns in N_2_O production that may be caused by variable O_2_ levels [32]–[37].

### Stable Isotope Dynamics of Gas Exchange

The 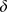 values of the net gas exchange flux are controlled by the kinetic fractionation factors for evasion (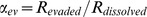, 0.9993 for 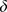 ^15^N and 0.9981 for 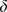 ^18^O) and invasion (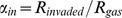, 1.0000 for 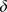 ^15^N and 0.9992 for 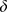 ^18^O) [22]. These two 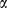 values are related to the equilibrium fractionation factor: 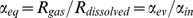 (0.99925 for 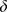 ^15^N and 0.99894 for 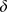 ^18^O) and are independent of temperature over the range of 0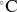 to 44.5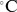 [22].

The 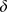 values of tropospheric N_2_O are 6.72 ‰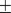 0.12‰ for 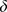 ^15^N and 44.62‰ 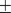 0.21‰ for 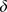 ^18^O [38]. Therefore, at equilibrium, dissolved N_2_O has dissolved 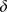 values slightly greater than these at 7.48‰ and 45.73‰, respectively.

In the model, net N_2_O flux between the atmosphere and dissolved phase was calculated using the thin boundary layer approach as:
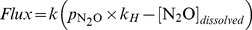
(3)where the N_2_O flux is calculated in mol/m^2^/h, 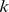 is the user-modifiable gas exchange coefficient (m/h), is the partial pressure of tropospheric N_2_O (assumed to be 320 ppbv from data provided by the ALE GAGE AGAGE investigators, [39], [40]), 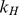 is the Henry constant for N_2_O (mol/atm-m^3^), and 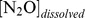 is the dissolved concentration of N_2_O (mol/m^3^). 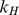 is a function of water temperature [41]:
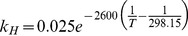
(4)where 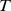 is temperature in kelvins.

Gas exchange is a two-way process. The net N_2_O flux rate (the difference between the invasion and evasion rates) depends on the dissolved N_2_O concentration. When a solution is at equilibrium with the atmosphere, the invasion and evasion rates will be equal, and the net flux will be zero.

As with the bulk N_2_O flux, the flux of the heavy isotopologues (^15^N_2_O and N_2_^18^O) can be calculated by including the kinetic fractionation factors for N_2_O (adapted from [27]):
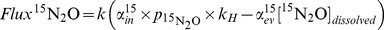
(5)




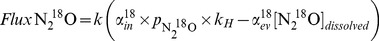
(6)where 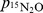 and 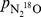 are the partial pressures of ^15^N_2_O and N_2_^18^O.

## Results

### Test of Model Performance

To test the ability of SIDNO to reproduce observed isotopic data, input parameters (N_2_O production rate, N_2_O 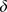 values, and 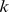) were set to replicate a series of experiments designed to derive fractionation factors for N_2_O gas exchange [22]. In these experiments, degassed water was exposed to N_2_O gas of known isotopic ratios in a sealed container to varying degrees of saturation.

**Figure 1:**
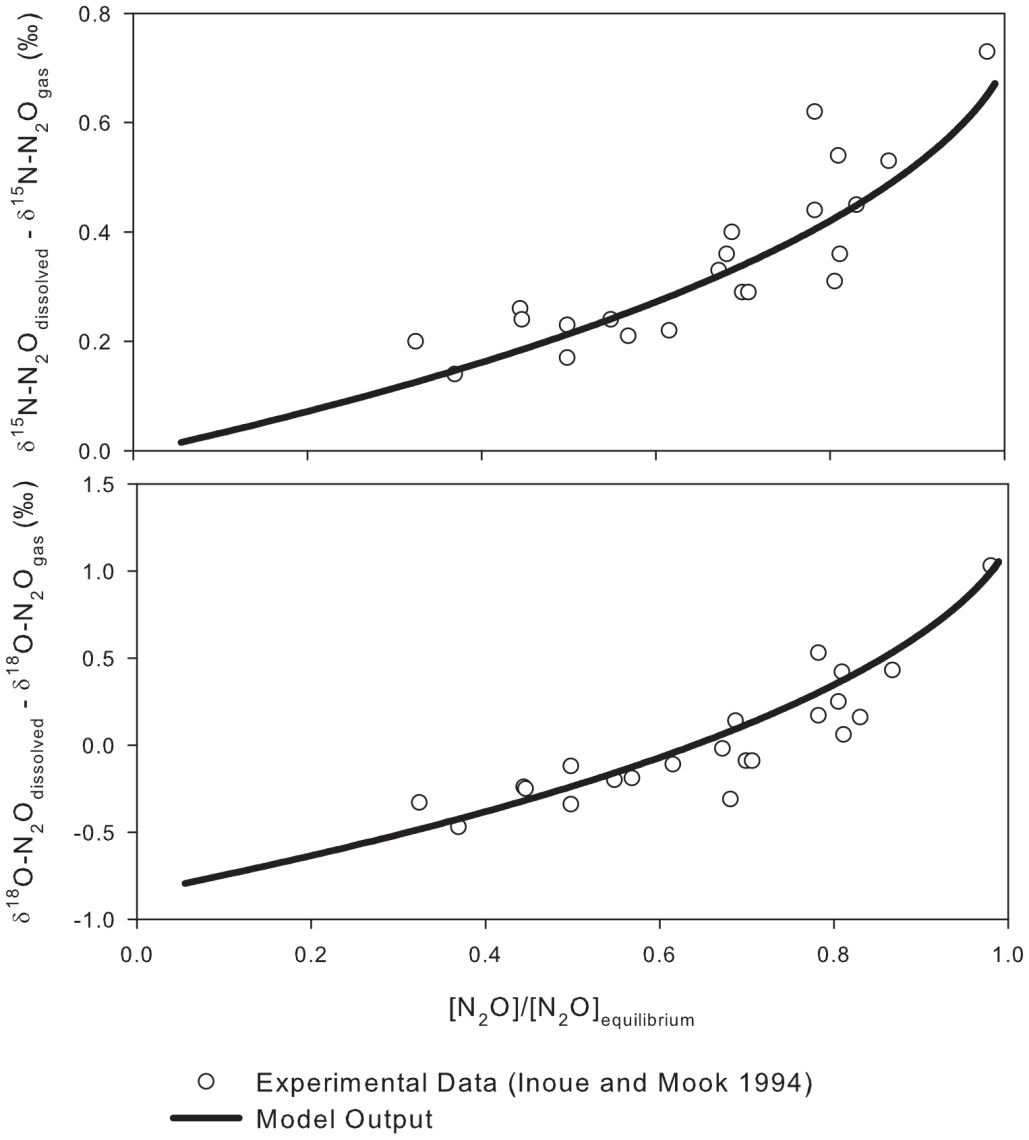
Comparing the model output to the experimental data of [22]. The coefficient of determination for experimental data and SIDNO model outputs were comparable to those of [22], 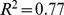 for 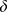 ^15^N and 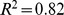 for 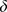 ^18^O. Precision of measurements for the experimental data was 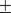 0.05‰ for 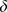 ^15^N and 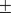 0.1‰ for 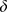 ^18^O.

Modelled dissolved N_2_O concentration and 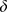 values increased in response to gas exchange (Figure 1). The model fit to the experimental data is comparable to the original best-fit derivations (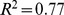 for 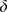 ^15^N and 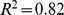 for 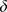 ^18^O for both the original fit [22] and the SIDNO fit) (Figure 1). The initial isotopic composition of dissolved N_2_O was identical to the gas phase 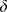 ^15^N value, but the 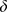 ^18^O of dissolved N_2_O was slightly less than the gas phase 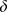 ^18^O value. Ultimately, at 100% saturation the 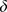 values of the dissolved N_2_O were greater than those of the gas phase as a result of 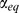. The model successfully simulated the kinetic and equilibrium fractionations during gas exchange under the experimental conditions.

**Figure 2:**
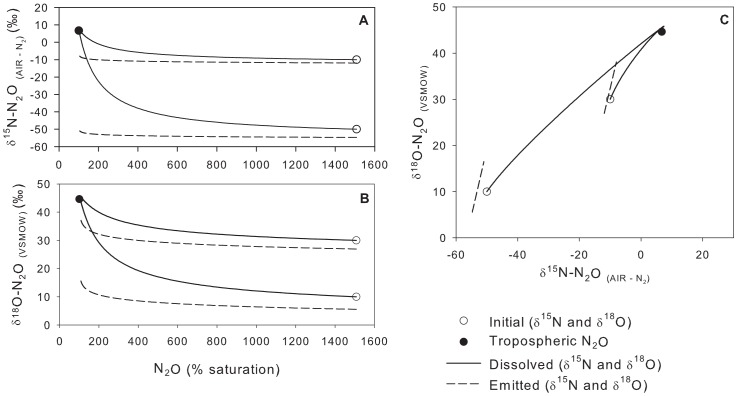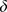 ^15^N and 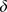 ^18^O trajectories for dissolved and emitted N_2_O in two supersaturated solutions with zero N_2_O production. Initial dissolved isotopic values for the two dissolved N_2_O solutions were 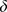 ^15^N = −50‰, 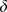 ^18^O = 10‰, and 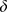 ^15^N = −10‰, 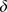 ^18^O = 30‰. Both runs used an initial dissolved N_2_O concentration of 1500% saturation. Note that in the 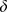 ^18^O versus 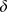 ^15^N plot, the dissolved N_2_O curves do not pass through the tropospheric N_2_O value due to the small equilibrium isotope effect.

Next, SIDNO was used to provide insight into the effect of degassing on the 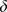 values of dissolved and emitted N_2_O. Here the results of two model runs with the same initial N_2_O concentration but different initial 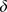 values of dissolved N_2_O were compared (Figure 2). As N_2_O saturation declined both the dissolved 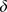 ^15^N values and instantaneous 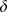 ^15^N values of the emitted N_2_O remained relatively constant, dissolved 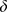 ^18^O values and instantaneous 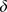 ^18^O values of the emitted N_2_O varied by about 10, when the solution was very supersaturated (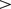
300% saturation). The 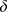 values rose quickly as the system approached 100% saturation. Because the light isotopologue diffuses out of solution faster than the heavy isotopologue, the instantaneous 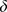 values of the emitted N_2_O were always less than the concomitant 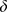 values of dissolved N_2_O. The isotopologues of N_2_O reached equilibrium independently of each other and therefore the total mass emitted for each isotopologue and rate of change depended on the initial concentration and 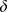 values. The retention of N_2_O in the dissolved phase caused the 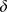 values of the mass emitted to differ from those of total mass production. However, when initial dissolved N_2_O concentrations were high (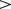
1000% saturation) the 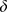 values of the total N_2_O emitted were similar to the 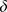 values of dissolved N_2_O because the mass of N_2_O lost is very much larger than the N_2_O that remained dissolved. The value of 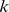 did not affect the gas exchange trajectories only the speed at which the system reached equilibrium.

N_2_O isotope data are often plotted as 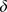 ^18^O versus 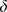 ^15^N to elucidate relationships between the various sources and tropospheric N_2_O [38]. The trajectories on these plots (Figure 2C) were dictated by the 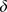 values of the source relative to the constant atmospheric value and the 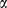 values. Note that some plots in the literature differ due to different reference materials for the 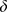 ^18^O scale (VSMOW and atmospheric O_2_).

### Modelling Scenarios with Steady State Production of N_2_O

The SIDNO model can be used to probe the stable isotope dynamics of N_2_O in a variety of situations that may be encountered in aquatic environments to elucidate the relationship between the N_2_O source (a function of N cycling processes), dissolved (the easily measured component), and emitted (of consequence for greenhouse gas production and global N and N_2_O cycle).

**Figure 3:**
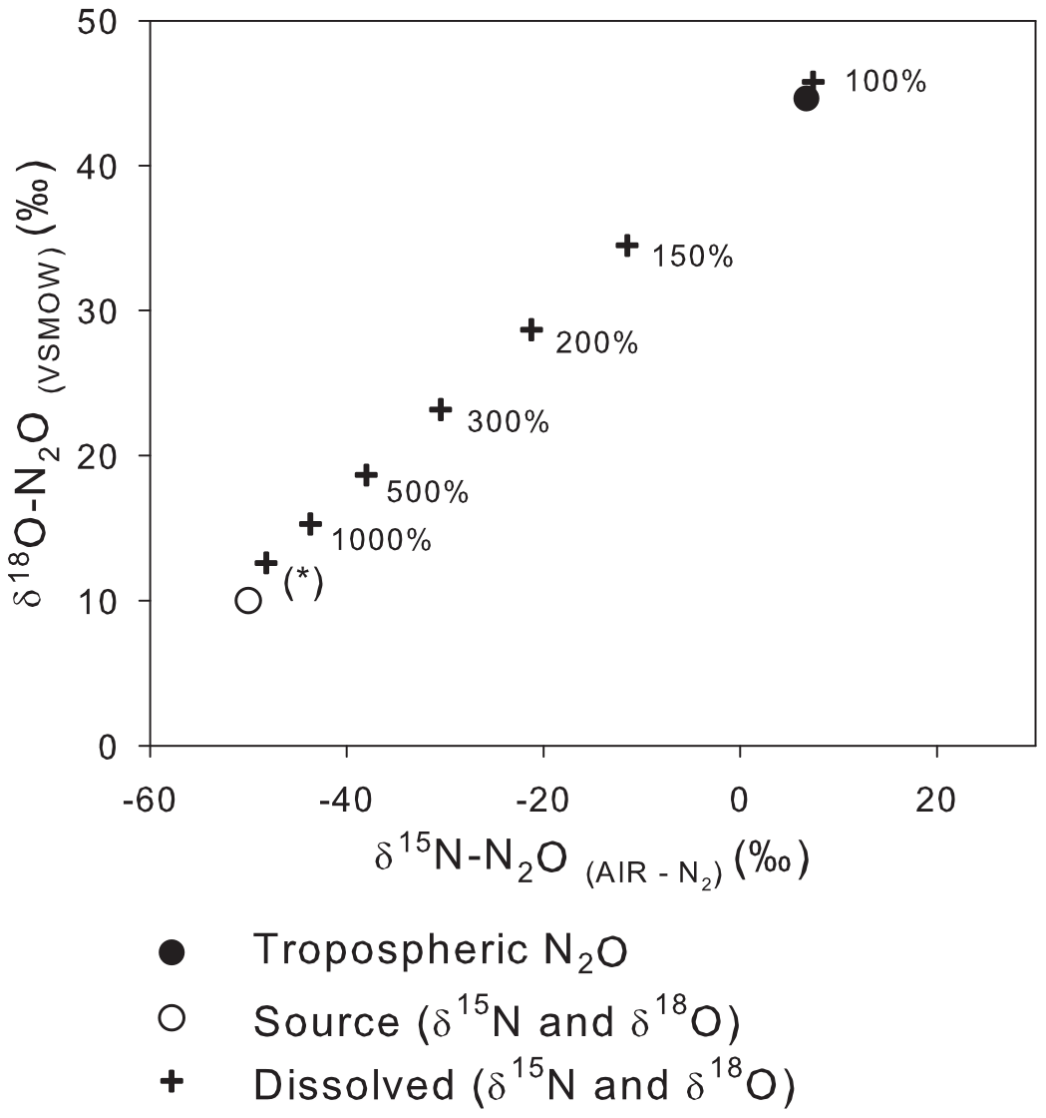
The relationship between 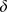 ^15^N, 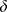 ^18^O and N_2_O concentration in a system at steady state with constant N_2_O production and open to gas exchange with the atmosphere. The point marked with a 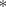 represents the minimum difference between the isotopic composition of dissolved and source N_2_O. The point at 100% saturation is the equilibrium value, the 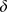 ^15^N and 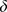 ^18^O of this point is controlled by the isotopic composition of tropospheric N_2_O and the equilibrium enrichment factors.

In the steady-state production of N_2_O (constant rate and 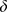 values), by definition, the 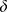 values of N_2_O production must be the same as those of the emitted N_2_O. As a result, the 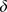 values of the dissolved N_2_O cannot equal that of the source (or emitted) N_2_O at steady state because the dissolved N_2_O must be offset from the emitted N_2_O by at least the 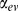 values. As the steady-state production rate was increased, the steady-state N_2_O concentration increased and the dissolved 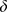 values approached but did not equal the source (Figure 3). Even at moderate supersaturations (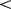
1000%) the effect of atmospheric N_2_O equilibration on the 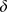 values of dissolved N_2_O cannot be ignored.

At steady state, the 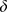 values of the emitted N_2_O must be equal to the source; the large difference between source/emitted and dissolved N_2_O underscores the importance of adjusting the measured 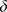 values of dissolved N_2_O in order to determine aquatic contributions of N_2_O to the atmosphere or N_2_O sources. This is critical when using dissolved measurements of N_2_O to constrain the global isotopic N_2_O budget, but not been done in most studies, e.g., [16], [42]–[44] but see [45].

### Modelling Scenarios with Variable Production of N_2_O

The relationship between the 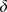 values of source, dissolved, and emitted N_2_O are much more complicated when N_2_O production is variable rather than when it is constant. N_2_O production may vary with respect to production rate and/or 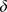 values; in many aquatic environments, N_2_O production is not likely to be constant. The N_2_O production processes, nitrification and denitrification, are sensitive to redox conditions, which can be highly variable, due to diel changes in dissolved O_2_ concentration, flow regime, etc. For example, [34] observed diel changes in the denitrification rate in the Iroquois River and Sugar Creek (Midwestern USA) and found that the denitrification was consistently greater during the day than night. The relative importance of nitrification and denitrification can change in response to the diel oxygen cycle: e.g., [46] observed a change from daytime nitrification to nighttime denitrification in a subtropical eutrophic stream. Coupling of N_2_O and O_2_ diel cycles has been observed in agricultural and waste-water treatment plant (WWTP) impacted rivers [36]. Since fractionation factors and substrates are different for nitrification and denitrification, ecosystem-scale fractionation factors may be rate and process dependent, and the 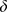 values of N_2_O production in a given ecosystem may not be constant over a diel cycle.

To simulate the diel variability, various scenarios were modelled by adjusting either production rate and/or the associated 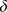 values. The variabilities in these input parameters were driven by a sine function with a 24 h period similar to a dissolve O_2_ curve. In all scenarios, the chosen range of production rates was based on published N_2_O flux rates (Table 1) and varied from 1 to 5 mol/m^2^/h^1^ (Table 2), which was between the diel variation in N_2_O flux observed by [33] and [46]. Temperature was held constant at 20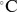. The value of 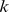 was varied between 0.1 and 0.3 m/d (Table 2), within the range observed in other river studies (Table 1). The combination of production rates and 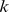 values were chosen to produce N_2_O between 150% and 500% saturation (Table 2) coinciding with the range of published data (Table 1). The range of 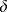 values used for the N_2_O source (Table 2) was within published values from various field studies [47]. For scenarios where the 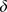 values of source N_2_O was variable, the sine function for the 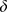 values was synchronized so that maxima and minima 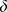 ^15^N values coincided with those of 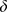 ^18^O. This was done for simplicity, and because, in general, nitrification yields N_2_O with lower 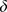 ^15^N and 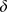 ^18^O values than denitrification (e.g., [10], [48]). Nevertheless, scenarios with greater amounts of N_2_O reduction to N_2_ can be modelled by increasing the source 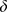 ^15^N and 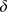 ^18^O values to those appropriate for any given ecosystem. Model scenarios were run until the output parameters (i.e., N_2_O saturation and the 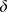 values of dissolved, source, and emitted N_2_O) reached dynamic steady state: model output was not constant over 24 h but the diel patterns on successive days were repeated.

**Table 1:** Summary of relevant published data on N_2_O production in aquatic environments.

Location	Range of N_2_O Saturation (%)	Range of N_2_O Flux ( 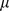 mol/m^2^/h^1^)	Range of 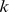 values (m/h)	Reference
Ohio River, OH, US	95 to 745	5 to 90	—	[12]
5 agricultural streams, ON, CA (over 2–3 years)	14 to 1700	−1 to 91.7	0.002 to 0.59	[5]
10 agricultural streams, ON, CA (over 17 diel cycles)	30 to 2570	−0.33 to 52.1	0.004 to 0.30	[45]
Bang Nara River, TH	170 to 2000	—	—	[20]
LII River, NZ	201 to 404	1.35 to 17.9	0.13 to 0.82	[58]
LII River, NZ	402 to 644	0.46 to 0.89	14.76	[33]
Seine River, FR	—	2.2 to 5.2	0.04 to 0.06	[59]
Canal Two, Yaqui Valley, MX	100 to 6000	0 to 34.9	0.3 to 0.6	[46]
agricultural stream, UK	100 to 630	0 to 37.5	—	[60]
Grand River, ON, CA	38 to 8573	−1.4 to 173.6	0.06 to 0.35	[36], [37]

**Table 2:** Summary of input parameters for the SIDNO model scenarios for non-steady state production of N_2_O.

Scenario #	Results Figure	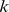	N_2_O Source Production Rate	N_2_O Source 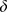 ^15^N and 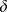 ^18^O Values
		(m/k)	( 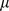 mol/m^2^/h^1^)	(‰)
*Variable Production Rate, Constant Isotopic Composition of Source*				
1	4	0.3	1 to 5	−50, 10
*Constant Production Rate, Variable Isotopic Composition of Source*				
2	5	0.3	3	−50, 10 to −30, 10
3	6	0.1	3	−50, 10 to −30, 10
*Variable Production Rate, Variable Isotopic Composition of Source*				
4*	7	0.3	1 to 5	−50, 10 to −30, 10
5**	8	0.3	1 to 5	−50, 10 to −30, 10
6*	9	0.1	1 to 5	−50, 10 to −30, 10

*Maximum production rate coincides with the lowest source 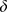 values.

**Maximum production rate coincides with the highest source 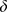 values.

### Model Scenario #1: Variable Production Rate, Constant Isotopic Composition of Source

In scenario #1 (Table 2), the 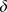 values of source N_2_O were held constant and the production rate was variable. An example of such a system may be N_2_O production via denitrification in river sediments with abundant 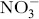. Denitrification rates in rivers have been observed to fluctuate in response to the diel O_2_ cycle [34]. If the fractionation factors for denitrification are not rate dependent, the resulting N_2_O production rate would be variable but the source 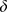 values of N_2_O values could be constant.

**Figure 4:**
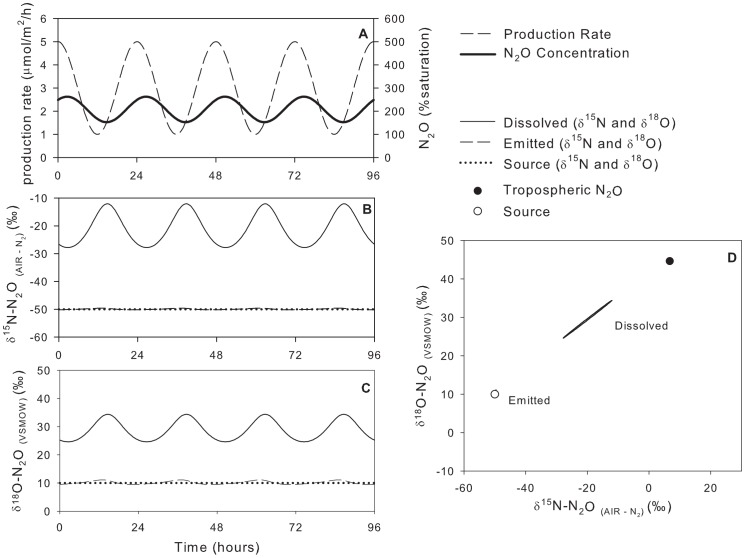
Model scenario #1 – isotopic composition of dissolved and emitted N_2_O with a variable production rate and constant isotopic composition of the source. Note, in panel D, the data points for emitted N_2_O are masked by the data point for source N_2_O.

Here, the maximum concentration lagged approximately 2.75 h behind the maximum N_2_O production rate, a function of the magnitude of the gas exchange coefficient, cf. [49]. The 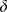 values for the instantaneously emitted N_2_O were relatively constant and very similar to the N_2_O source (within 0.4‰ for 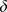 ^15^N and 1.1‰ for 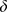 ^18^O, Figure 4, Table 3). However, the 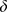 values of dissolved N_2_O were more variable, spanning 16‰ for 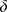 ^15^N and 10‰ for 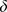 ^18^O. Thus, a change in the 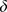 values of dissolved N_2_O can be driven simply by a change in production rate and not necessarily a change in the 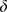 values of the source. Since the system was at dynamic steady state, the average 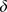 values of the emitted N_2_O were identical to the average 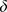 values of the source. This must be true in all steady-state cases to conserve the mass of the N_2_O isotopologues.

**Table 3:** Summary of SIDNO output for model scenarios simulating non-steady state production of N_2_O.

	Dissolved N_2_O				Emitted N_2_O		
Scenario #	Saturation	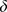 ^15^N, 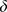 ^18^O	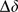 ^15^N	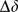 ^18^O	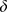 ^15^N, 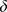 ^18^O	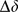 ^15^N	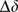 ^18^O
	(%)	(‰)	(‰)	(‰)	(‰)	(‰)	(‰)
*Variable Production Rate, Constant Isotopic Composition of Source*							
1	153 to 263	−27.8, 24.6 to −12.1, 34.4	37.9	24.4	−49.6, 9.5 to −50.2, 11.1	0.2	0.5
*Constant Production Rate, Variable Isotopic Composition of Source*							
2	208	−19.6, 29.4 to −3.7, to 37.4	30.4	19.4	−45.3, 12.3 to −14.7, 27.7	4.7	2.3
3	423	−26.1, 24.8 to −15.1, 30.3	23.9	14.8	−37.2, 16.4 to −22.8, 23.6	12.8	6.4
*Variable Production Rate, Variable Isotopic Composition of Source*							
4*	153 to 263	−25.8, 25.6 to −1.2, 39.8	24.2	15.6	−46.9, 11.3 to −18.0, 26.5	8	3.5
5**	153 to 263	−11.2, 33.8 to −5.0, 36.1	38.8	23.8	−41.6, 14.6 to −13.4, 28.1	8.4	4.6
6*	345 to 501	−31.6, 21.6 to −18.4, 29.3	18.4	11.6	−42.1, 13.7 to −29.4, 20.6	19.4	9.4

Temporally variable parameters are given as a range. 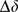 ^15^N and 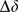 ^18^O (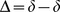) are the maximum difference between the range of source N_2_O and the range for the model output parameter.

*Maximum production rate coincides with the lowest source 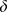 values.

**Maximum production rate coincides with the highest source 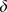 values.

In some aquatic systems, the N_2_O production rate may remain constant with time but the 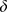 values of the source may change with time. In rivers or lakes without a strong diel O_2_ cycle, sediment denitrification may produce N_2_O at an approximately constant rate. Denitrification rate may also be independent of water column 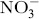 concentration if limited by factors other than diffusion in the sediments. The 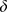 values of the source N_2_O may thus change if the 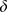 values of the 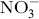 substrate changed with time. For example, many studies have shown that the 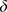 values of residual 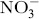 increase during denitrification [50]. Similarly, 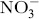 from WWTPs may have different 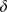 values than agricultural runoff and diel changes in WWTP release may result in changing 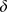 values of 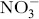. Changes in N cycling may also vary on a diel basis but result in fortuitously similar N_2_O production rates due to, for example, changes in the N_2_O:N_2_ ratio of denitrification or changes in the relative importance of nitrification and denitrification. Thus changes in 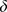 values of the N_2_O source do not necessarily indicate changes in N_2_O production rates.

### Model Scenario #2: Constant Production Rate, Variable Isotopic Composition of Source

**Figure 5:**
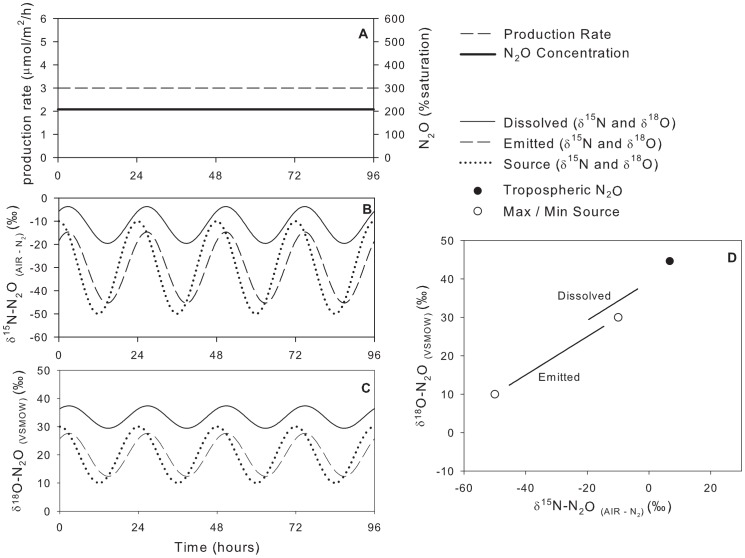
Model scenario #2 – Isotopic composition of dissolved and emitted with a constant production rate and variable isotopic composition of the source.

In scenario #2, when the N_2_O production rate was held constant and the 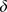 values of the source varied with time (from −50‰ to −10‰ for 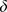 ^15^N and from 10‰ to 30‰ for 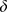 ^18^O), the 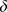 values of the dissolved N_2_O was also much farther from that of the source than the dissolved N_2_O due to the effects of atmospheric exchange and the emitted N_2_O varies linearly between the two source values. In contrast, the dissolved N_2_O is parallel but offset from the line connecting the two sources (Figure 5, Table 3). The maximum difference between emitted and source N_2_O was 4.7‰ for 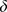 ^15^N and 2.3‰ for 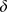 ^18^O. The dissolved and emitted 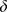 values also lagged 2.75 h behind the source as a result of gas exchange (as above). Since the system was at dynamic steady state, the average 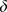 values of the emitted N_2_O were identical to the average 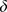 values of the source.

### Model Scenario #3: Constant Production Rate, Variable Isotopic Composition of Source

**Figure 6:**
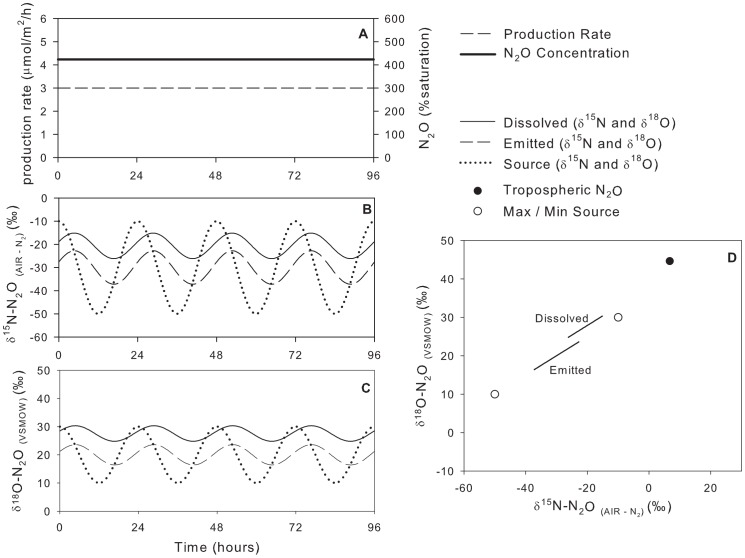
Model scenario #3 – Isotopic composition of dissolved and emitted N_2_O with a constant production rate and variable isotopic composition of the source. 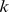
 is reduced from 0.3 m/h to 0.1 m/h.

To examine the effects of varying 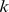 on the scenario of constant N_2_O production with variable isotopic signature of the source, 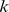 was reduced from 0.3 m/h (scenario #2) to 0.1 m/h (scenario #3; Figure 6, Table 3). The 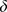 values for the emitted N_2_O were centred between the sources N_2_O values, but dissolved N_2_O 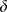 values were farther from tropospheric N_2_O than the high-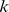 scenario #2 (Figure 6 D).

The effect of reducing 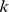 was an increase in N_2_O concentration with the same production rate and a shift in the 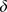 values of dissolved N_2_O toward the source values. Reducing 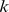 also dampened the response between the instantaneous 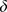 values of the emitted N_2_O and the 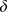 values of the source. As above, the lag time between the 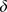 values of the source and emitted N_2_O increased as 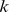 decreased. The total range of the source and emitted 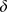 values decreased. The difference between the source and emitted 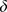 values was 12.8‰ for 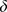 ^15^N and 6.4‰ for 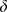 ^18^O.

To simulate a system alternating between two N_2_O production processes, such as differing relative contributions of nitrification and denitrification, with different rates of N_2_O production and 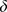 values, the model was run with both production rate and its 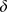 values variable with time (scenarios #4, #5, and #6). The production rate and 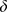 values were adjusted so that the maximum rate coincided with the lowest source 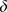 values in scenarios #4 and #6 and so that maximum rate coincided with the highest source 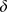 values in scenario #5.

### Model Scenario #4: Variable Production Rate, Variable Isotopic Composition of Source

**Figure 7:**
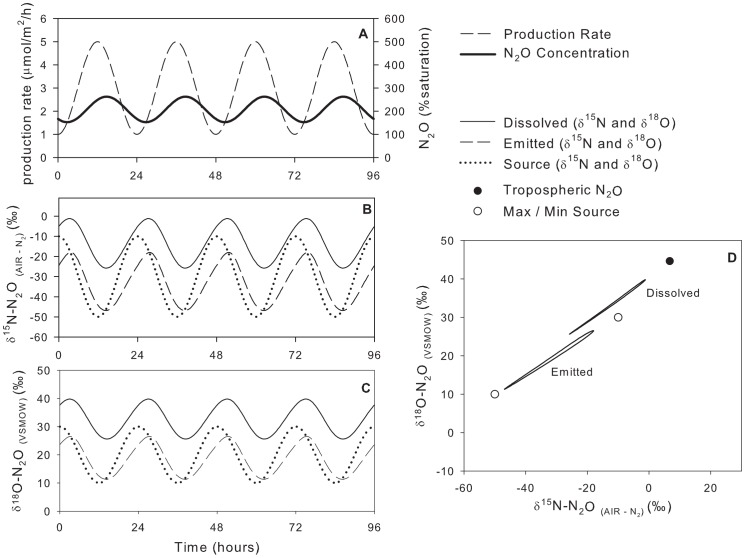
Model scenario #4 – Isotopic composition of dissolved and emitted N_2_O with a variable production rate and variable isotopic composition of the source. Maximum production rate is in sync with the lowest 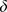 ^15^N and 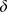 ^18^O values of the source.

For scenario #4, the resulting N_2_O concentrations were identical to those in model scenario #1, with the maximum concentration lagging approximately 2.75 h behind the maximum production rate (Figure 7, Table 3). The relationship between the 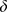 values of the dissolved and emitted N_2_O was more complex than in other scenarios. The lag time between the maximum source 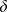 values and those of dissolved and emitted N_2_O (when the production rate was minimum) was 3.75 h; however, the lag time between the minimum source 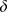 values and those of the dissolved and emitted N_2_O (when the production rate was maximum) was only 2.25 h. The difference between the emitted and source N_2_O was 3.1‰ to 8.0‰ for 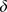 ^15^N and 1.3‰ to 3.4‰ for 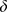 ^18^O. The 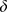 values of emitted N_2_O were closer to those of the source during periods of high production rates (and thus higher concentrations) than periods of low production rates. However, the flux-weighted average 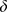 values of emitted N_2_O were equal to the average production-weighted source 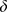 values because the system was at dynamic steady state.

### Model Scenario #5: Variable Production Rate, Variable Isotopic Composition of Source

**Figure 8:**
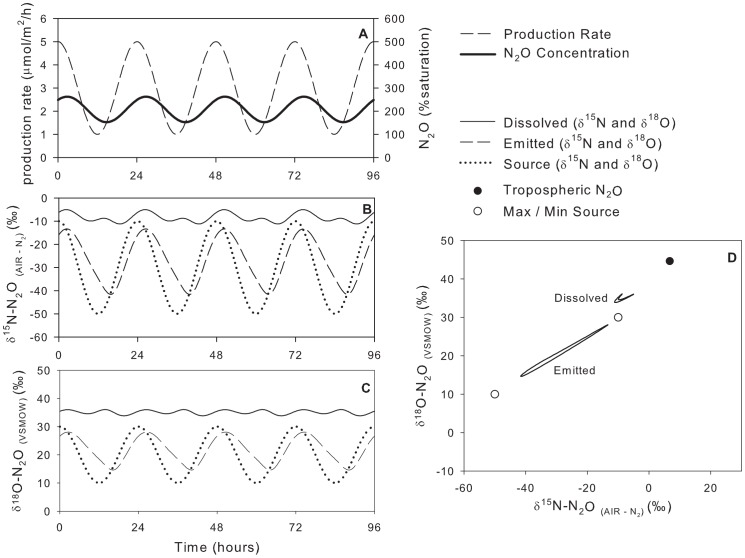
Model scenario #5 – Isotopic composition of dissolved and emitted N_2_O with a variable production rate and variable isotopic composition of the source. Maximum production rate is in sync with the greatest 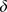 ^15^N and 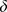 ^18^O values of the source.

The isotopic counterpoint to scenario #4 is adjusting the timing of maximum N_2_O production to coincide with the highest 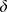 values of production (scenario #5). All other parameters were the same as scenario #4 (Table 3). The resulting pattern for the 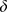 values of dissolved N_2_O was very different than scenario #4 (Figure 8, Table 3). While the dissolved N_2_O concentrations were identical to the model scenario #4, the 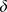 values of dissolved N_2_O were nearly constant with time. The relationship between the 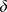 values of emitted and source N_2_O was similar to scenario #4, although the instantaneous difference in 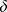 values were slightly greater. The 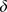 values of the dissolved N_2_O were greatly dampened by the fact that maximum production rate coincided with source 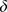 values that were closest to tropospheric N_2_O. In scenario #4, the high rates of N_2_O production at 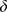 values very different than tropospheric N_2_O increased the amplitude of the 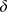 values of dissolved N_2_O.

### Model Scenario #6: Variable Production Rate, Variable Isotopic Composition of Source

**Figure 9:**
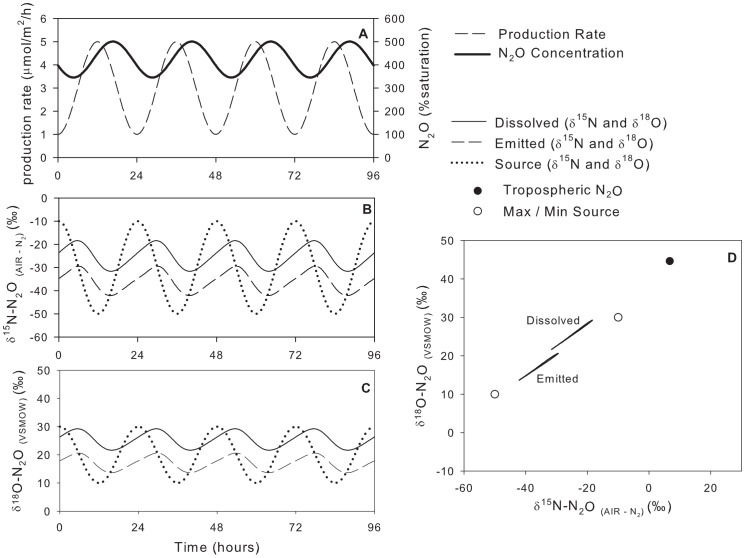
Model scenario #6 – Isotopic composition of dissolved N_2_O and emitted N_2_O with a variable production rate and variable isotopic composition of the source. Maximum production rate is in sync with the lowest 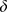 ^15^N and 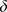 ^18^O values of the source. 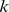 is reduced from 0.3 m/h to 0.1 m/h.

To determine the effects of a lower 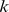 on model scenario #4, 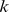 was reduced from 0.3 m/h from 0.1 m/h for scenario #6. As shown above, lower 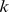 increased the dissolved N_2_O concentrations and dampened the diel range of 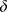 values of both dissolved and emitted N_2_O (Table 3, Figure 9). Lower 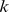 also increased the lag time between the 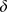 values of emitted and source N_2_O and increased the difference between the 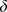 values of emitted and source N_2_O (Figure 9). As in all scenarios, the flux-weighted average 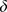 values of emitted N_2_O were equal to the average production-weighted source 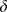 values.

### Grand River

The ability of SIDNO to reproduce measured patterns of N_2_O concentration and 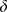 values in a human-impacted river was also assessed. The Grand River is a seventh-order, 300 km long river that drains 6800 km^2^ in southern Ontario, Canada, into Lake Erie, see [36], [37], [51]. There are 30 WWTPs in the catchment and their cumulative impact can be observed via the increase in artificial sweeteners in the river [52].

Samples were collected approximately hourly for 28 h at two sites in the central, urbanized portion of the river: sites 9 and 11 in [51], [52]. The upstream site, Bridgeport, is where the river enters the urban section of the river at the city of Waterloo and is immediately above that city's WWTP. Blair is 26.6 km downstream of Bridgeport and below the cities of Waterloo and Kitchener. It is also 5.5 km downstream of the Kitchener WWTP. Average river depth at both sites was 30 cm. Values of 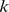 were determined by best-fit modelling of diel O_2_ and 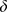 ^18^O-O_2_ values at the sites [36]. N_2_O concentration analyses were performed on a Varian CP-3800 gas chromatograph with an electron capture detector and isotopic ratio analyses were performed on a GV TraceGas pre-concentrator coupled to a GV Isoprime isotope ratio mass spectrometer, see [5] for analytical details.

**Figure 10:**
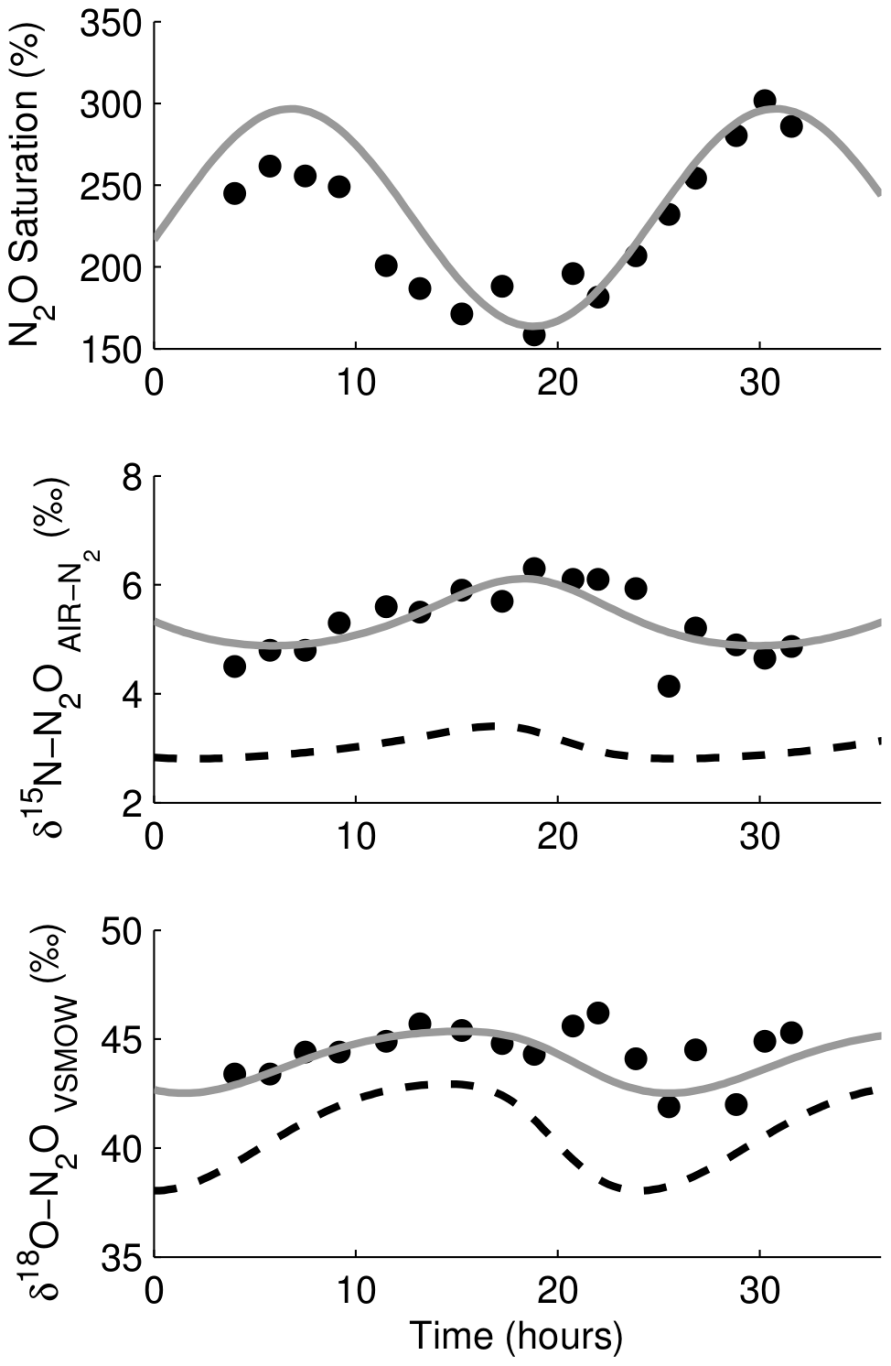
Diel variability in N_2_O concentration and 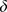 values at Bridgeport in the Grand River, Canada. The time axis begins at 00∶00 on 2007-06-26. Maximum production rate is in sync with the greatest 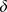 ^18^O values of the source, while 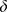 ^15^N of the source was constant. 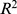 values between field and model data for N_2_O saturation, 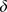 ^15^N, and 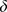 ^18^O values are 0.83, 0.68, and 0.30. This is similar to model scenario #4 (Figure 7).

**Figure 11:**
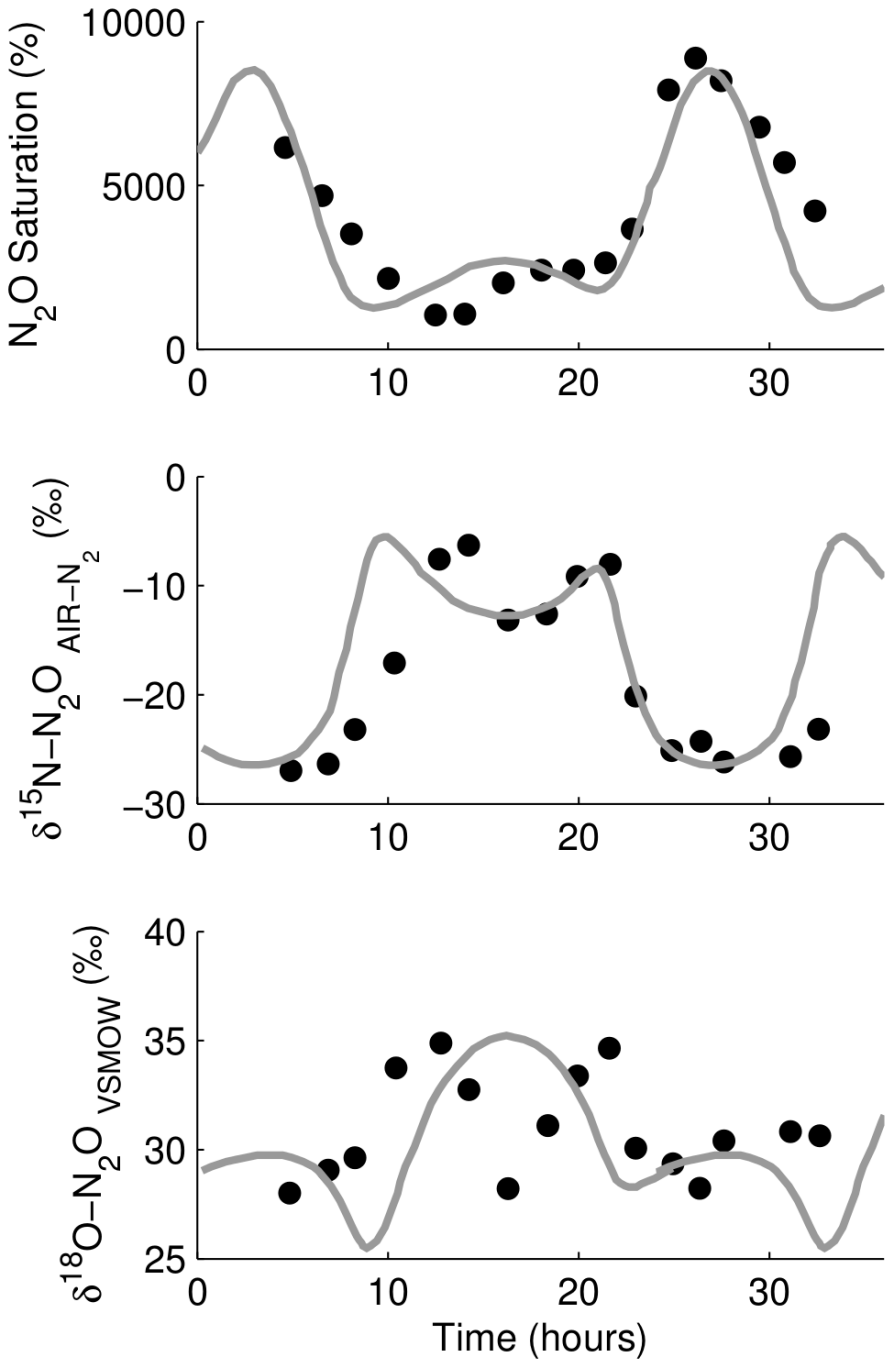
Diel variability in N_2_O concentration and 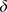 values at Blair in the Grand River, Canada. The time axis begins at 00∶00 on 2007-06-26. Maximum production rate is in sync with the lowest 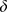 ^15^N and 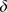 ^18^O values of the source. 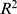 values between field and model data for N_2_O saturation, 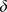 ^15^N, and 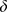 ^18^O values are 0.78, 0.53, and 0.03. This is similar to model scenario #5 (Figure 8).

Data from upstream and downstream of large urban waste-water treatment plants on the Grand River show diel patterns in N_2_O saturation and 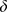 values (Figures 10 and 11). At the Bridgeport site, the diel patterns of N_2_O saturation and 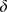 ^15^N values were opposite of each other, that is, when N_2_O saturation was highest around sunrise the 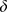 ^15^N values were lowest and when when N_2_O saturation was lowest around before sunset the 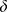 ^15^N values were greatest. 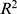 values between field and model data for N_2_O saturation, 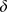 ^15^N, and 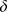 ^18^O values are 0.83, 0.68, and 0.30. Model results reproduce the range and sinusoidal patterns of the field data though the 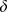 ^18^O fit was poor in the second half of the field data. The diel pattern in 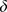 ^18^O values was similar to that of 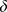 ^15^N but was shifted earlier by about 4 h. These patterns were similar to those of scenario #4 (variable N_2_O production and variable 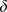 values of the source N_2_O coinciding when maximum production rates coincided with lowest source 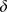 values) and the result of consistent diel five-fold variability in N_2_O production and variability in the 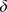 ^18^O of the N_2_O produced in the river.

At the downstream Blair site, both 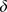 ^15^N and 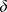 ^18^O values were much lower and exhibited a greater range than at Bridgeport. 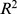 values between field and model data for N_2_O saturation, 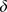 ^15^N, and 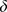 ^18^O values are 0.78, 0.53, and 0.03. Model results reproduce the range and peak-and-trough pattern of the N_2_O saturation and 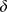 ^15^N data. Model results reproduce the range of 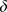 ^18^O values but the pattern is not well reproduced. While all data at Bridgeport exhibited smooth, sinusoidal diel changes, the data at Blair show rapid changes. The diel patterns of N_2_O saturation and 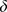 ^15^N values were opposite of each other, that is, when N_2_O saturation was highest around midnight, the 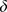 ^15^N values were lowest and when when N_2_O saturation was lowest during mid-day, the 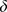 ^15^N values were greatest. The diel pattern in 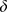 ^18^O values was more complex at Blair than at Bridgeport suggesting that daytime and nighttime were associated with different 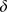 ^18^O values of N_2_O production. These patterns were similar to those of scenario #5 (variable N_2_O production and variable 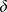 values of the source N_2_O coinciding when maximum production rates coincided with highest source 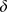 values) and the result of a five-fold variability in day-to-night N_2_O production and variability in 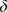 ^15^N and 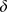 ^18^O of the N_2_O produced in the river.

For both Bridgeport and Blair data, the cause of poorer fits for 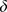 ^18^O than 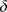 ^15^N deserve further research. Adding concomitant measurements of 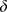 ^15^N and 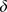 ^18^O values of 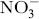 may provide clues about N cycling and help explain some of the observed variability in N_2_O [53]. Predicting 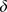 ^18^O-N_2_O values from nitrification [11] and denitrification [54] is difficult because of the complex relationship between 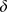 ^18^O-H_2_O values and 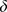 ^18^O-N_2_O values. Additionally, diel variability in N_2_O reduction to N_2_ [45], [46], may also manifest itself in 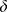 ^18^O-N_2_O values because of the strong O isotope fractionation factor during denitrification [55].

## Discussion

Calculating the 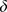 values of emitted or source N_2_O is critical for regional and global N_2_O isotopic budgets and also provides information about the source of N_2_O and thus N cycling processes. However, SIDNO can simulate the relationships between the 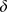 values of dissolved, source, and N_2_O emitted from aquatic ecosystems to the atmosphere.In systems with N_2_O production at dynamic steady state, the 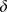 values of dissolved N_2_O will not always be directly indicative of the 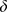 values of the source N_2_O. The difference between dissolved and source 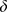 values increases as N_2_O saturation decreases (as demonstrated in Figures 2 and 3). Even above 1000% saturation (from high production rates and/or low 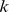), the 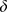 values of dissolved N_2_O will only approach 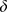 values of the source but offset by 0.7‰ for 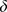 ^15^N and 1.9‰ for 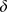 ^18^O, a result of the 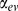 values (Figure 3). At constant N_2_O production rates and 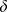 values, the source and emitted 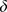 values can be quantified since the 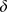 values of emitted N_2_O must be identical to those of the source and can be calculated from dissolved values (Figure 3; equations 5 and 6).

Our modelling results identified the limitations associated with simple interpretation of dissolved N_2_O isotope data since the 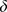 values of dissolved and emitted data are synchronous but rarely offset by a constant value. If N_2_O saturation changes with time, the N_2_O production rate must also have changed with time, provided 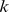 had been constant (compare model scenarios #1 and #2 in Figures 4 and 5). In contrast, changes in 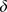 values of dissolved N_2_O do not require a change in the source 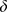 values (model scenario #1 and Figure 4), while constant 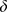 values of dissolved N_2_O do not require constant source 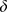 values (for example model scenario #5 in Figure 8).

When 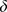 values of the source N_2_O are variable, the relationship between emitted and source N_2_O becomes complicated. The 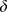 values of emitted N_2_O will lag behind those of the source and the amplitude of the diel range of 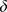 values will be dampened relative to the source. The amount of lag and dampening is a function of 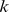, N_2_O production rate and timing, and the proximity of the source 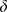 values to those of the atmosphere (compare Figures 2 with 3 and Figures 4 with 6). Qualitatively, the 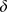 values of emitted N_2_O will be similar to the source if the equilibration time of dissolved N_2_O is small relative to the period of source variability (e.g., 24 h period due to diel changes in N cycling [36], [45]). Assuming homogeneous N_2_O release upstream, the equilibration time can be approximated from a decay curve as 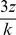, where 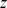 is mean depth [49]. If 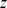 is small and/or 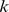 is high, the equilibration time will be short and the 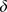 values of the emitted N_2_O will be close to the source. With decreasing 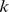 (or increasing equilibration time), the 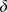 values of emitted N_2_O will lag farther behind and will always have a smaller range of 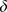 values than the source. At the most extreme case, the variability in the 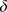 values of emitted N_2_O will be reduced to nearly zero and 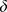 values of the emitted N_2_O would be equal to the average production-weighted source 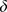 values. At very long equilibration times, the probability of N_2_O consumption increases, a process not explicitly included in SIDNO where the 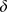 value of the source N_2_O is simply that which is released to the water column.

Separating N_2_O production into nitrification and denitrification requires independent knowledge about the 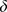 values of the source N and O in aquatic ecosystems. It is therefore not possible to state a single 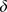 ^15^N value for nitrification–N_2_O and one for denitrification–N_2_O applicable to all aquatic ecosystems. The 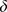 ^15^N value of the N_2_O precursors 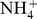 and 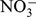 vary across ecosystem as a result of human impact and N loading (agricultural and WWTP) as well as the source of N, and additional N transformations in the aquatic ecosystem. For example, along the length of the Grand River, 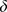 ^15^N values of 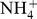 and 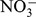 exhibit systematic trends resulting from the confluence of agricultural tributaries and large urban waste-water treatment plants (Schiff et al., unpublished results, [53]). Nevertheless, these values can be measured and biogeochemical relationships between N species, redox, and N_2_O can be used as supporting information for process separation (e.g., [5], [12], [36], [45]). The 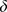 ^18^O value of N_2_O will also vary across ecosystems as a result of its close relationship with 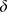 ^18^O-H_2_O and to a lesser extent 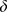 ^18^O-O_2_ [10], [11], [54], [56]. Fortunately, 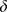 ^18^O-H_2_O values can be easily predicted and measured [57]. Thus, once 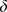 values of N_2_O precursors have been identified, biogechemical data can provide an indication about the diel pattern of N_2_O production processes, and ranges of potential end-member 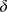 values can be calculated (e.g., [5] summarize isotopic fractionation for 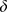 ^15^N and [10], [11], [54], [56] for 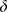 ^18^O) and the model used to fit the field data.

## Conclusions

In aquatic ecosystems, the instantaneous 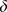 values of N_2_O emitted to the atmosphere are easily calculated if the water temperature and dissolved N_2_O concentration and 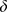 values are known. Our modelling efforts illustrate that complex relationships exist between dissolved and source N_2_O and that the 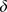 values of dissolved N_2_O are not always representative of either the N_2_O produced or emitted to the atmosphere. Thus, calculated 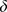 values of the emitted N_2_O are the values that should be used to draw conclusions about N_2_O emission from aquatic systems and the global N_2_O cycle rather than the more commonly used instantaneous values (Table 4). The flux-weighted 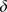 values of emitted N_2_O can provide average production-weighted 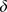 values of the N_2_O source under dynamic steady-state in aquatic ecosystems.

**Table 4:** Summary of the results of the SIDNO modelling as the predictive relationship between observations and implications.

Observed Parameter	Implications	Examples
Dissolved N_2_O concentration is constant with time	The N_2_O production rate is constant with time (if k and temperature are also constant). The N_2_O flux to the atmosphere is equal to the production rate. This may not be true if the concentration is close to atmospheric equilibrium.	Scenarios #2 and #3
Dissolved N_2_O concentration is variable with time in a sinusoidal pattern	The N_2_O production rate is variable with time (if concentration change cannot be explained by change in k or temperature). The average N_2_O flux to the atmosphere is equal to the average production rate.	Scenario #1
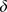 ^15^N and 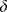 ^18^O of dissolved N_2_O is constant with time	The observation is inconclusive. At concentrations near atmospheric equilibrium, isotopic composition of dissolved N_2_O will approximate tropospheric N_2_O, egardless of source values. A constant isotopic signature of dissolved N_2_O that is different from tropospheric N_2_O can indicate either a constant source (if production rate is constant), or a variable source.	Scenario #5
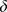 ^15^N and 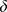 ^18^O of dissolved N_2_O is variable with time (slope of data on a 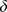 ^18^O– 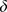 ^15^N cross-plot tends toward tropospheric N_2_O)	The change in 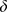 ^15^N and 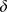 ^18^O of dissolved N_2_O is likely a result of a change in concentration, but it is possible that the source is variable with time, if the 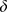 ^15^N and 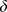 ^18^O values of the source also trend through the value for tropospheric N_2_O.	Scenarios #1, #2, #4, and #6
Calculated 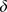 ^15^N and 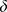 ^18^O of emitted N_2_O is constant with time	The isotopic composition of the source is constant with time, and equal to the calculated value for emitted N_2_O	Scenario #1
Calculated 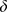 ^15^N and 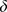 ^18^O of emitted N_2_O is variable with time	The isotopic composition of the source is variable with time. The range in 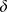 ^15^N and 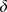 ^18^O of emitted N_2_O is the minimum for the range in that of the source. The flux weighted average 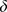 ^15^N and 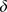 ^18^O of emitted N_2_O is equal to the production weighted average source values.	Scenarios #2–#6
Long residence time relative to variability of source (need to independently determine 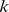 )	The changes in N_2_O concentration and 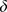 ^15^N and 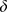 ^18^O of emitted N_2_O will be dampened relative to, and lag behind, that of the source	Scenarios #3 and #6
Short residence time relative to variability of source (need to independently determine 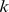 )	The changes in N_2_O concentration and 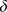 ^15^N and 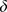 ^18^O of emitted N_2_O will be indicative of changes in the source	Scenarios #1, #2, #3 and #4

If the 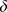 values of emitted N_2_O are constant with time, either the 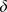 values of the source must also be constant or the N_2_O equilibration time is very long. However, if the calculated 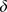 values of emitted N_2_O vary with time then the 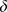 values of the source must also vary with time producing a diagnostic pattern. These findings are more robust than using dissolved 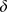 values alone since dissolved 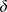 values can change simply with a change in N_2_O production rate, changes in source 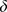 values, and changes in 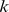. N_2_O residence time, dependent on production rate, 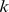, and 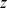, will determine the lag time between the 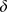 values of emitted and source N_2_O. The difference in timing between maxima and minima 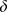 values of emitted N_2_O and the maxima and minima of dissolved N_2_O is indicative of how the 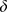 values of the source change. For all these reasons, we urge caution when using single samples of N_2_O concentration and 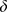 to calculate fluxes of N_2_O to the atmosphere and inferring N_2_O production pathways.

Ultimately, the dynamic model SIDNO may be used to estimate 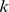, N_2_O production rate and 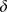 values of the N_2_O source, an indication of the production pathway and N cycling, in aquatic ecosystems via inverse modelling. If physical properties, such as depth and temperature are known, SIDNO may be used to fit the measured field data (N_2_O concentration and 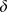 values) by adjusting the N_2_O source parameters. SIDNO can also be used to explore the dynamics between dissolved, source, and emitted N_2_O to query production scenarios and design field campaigns for studies of N cycling processes.
