# Correction: Proper Interpretation of Dissolved Nitrous Oxide Isotopes, Production Pathways, and Emissions Requires a Modelling Approach

**DOI:** 10.1371/journal.pone.0099549

**Published:** 2014-05-30

**Authors:** The PLOS ONE Staff

There is a missing superscript in the third sentence of the first paragraph of the Materials and Methods section. 15R should be “^15^R.”

Reference 50 is incorrect due to typesetting errors. The correct reference is:

Mengis M, Schif SL, Harris M, English MC, Aravena R, et al. (1999) Multiple Geochemical and Isotopic Approaches for Assessing Ground Water NO_3_^−^ Elimination in a Riparian Zone. Ground Water 37: 448–457. doi: 10.1111/j.1745-6584.1999.tb01124.x

Reference 53 is incorrect due to typesetting errors. The correct reference is:

Hood JLA, Taylor WD, Schiff SL (2013) Examining the fate of WWTP effluent nitrogen using *δ* ^15^N–NH_4_ ^+^, *δ* ^15^N–NO _3_ ^−^ and *δ* ^15^N of submersed macrophytes. Aquat Sci. doi: 10.1007/s00027-013-0333-4.
